# Blood Group Type Association with Head and Neck Cancer

**DOI:** 10.3390/hematolrep14010005

**Published:** 2022-03-02

**Authors:** Gaube Alexandra, Michire Alexandru, Calangiu Filip Stefan, Draghia Petruta-Maria, Burlacu Mihnea Gabriel, Georgescu Dragos-Eugen, Georgescu Mihai Teodor

**Affiliations:** 1 National Institute of Infectious Diseases “Prof. Dr. Matei Bals”, 021105 Bucharest, Romania; gaube_alexandra@yahoo.com; 2 Department 8 Radiology, Oncology, Hematology, “Carol Davila” University of Medicine and Pharmacy, Bulevardul Eroii Sanitari 8, 050474 Bucharest, Romania; gfdragos@yahoo.com (G.D.-E.); georgescumihaiteodor@gmail.com (G.M.T.); 3 Radiation Therapy Department, “Prof. Dr. Al. Trestioreanu” Oncology Institute, Sos. Fundeni No. 252, 022328 Bucharest, Romania; calangiufilip@yahoo.com (C.F.S.); dpetruta@gmail.com (D.P.-M.); mihnea.burlacu93@gmail.com (B.M.G.); 4 Clinical Hospital “Dr Ion Cantacuzino”, 030167 Bucharest, Romania

**Keywords:** blood group, hypopharyngeal cancer, oral cavity cancer, oropharyngeal cancer, protective factor

## Abstract

Background: We conducted an analysis to check whether the ABO blood group impacts the susceptibility or protection against different types of head and neck cancers. Method: We analyzed the medical records of 61,899 cancer patients from “Prof. Dr. Alexandru Trestioreanu” Institute of Oncology from Bucharest, along with the corresponding blood group type. Data were scraped using Python. For analysis, we used Chi-square test. Results: The blood group count was A (245, 45.12%) followed by 0 (160, 24.66%), B (110, 20.26%), and AB (28, 5.16%). Hypopharyngeal cancer was associated with B group, oral cavity cancer was associated with a lower risk in patients with B group while AB patients had a higher risk for oral cavity cancer (χ^2^ = 36.136, df = 18, *p* = 0.007). Conclusion: Blood group B is associated with an increased incidence for hypopharyngeal cancer, whereas, for the oral cavity, was associated lower incidence. Blood antigen A is associated with a higher risk of oral cavity cancer development, independent of B blood antigen.

## 1. Introduction

The ABO blood group systems antigens were discovered in late 1900 and are considered by many the first human genetic markers. [1] Over the last five decades, studies focusing on ABO antigens found that those are expressed on the surface of many other cells than red blood cells, including epithelial and endothelial cells [2]. This further led to research regarding an existing link between ABO antigens and chronic diseases, including malignancies [3,4,5]. It has been recently discovered that the existence of ABO antigens on the surface of cancer cells, although different from normal cell ABO antigens, is still associated with the ability of these cells to escape an immune system reaction, escaping apoptosis [6].

Recent research in medical oncology focused on different types of cancer, depending on their frequency, identifying a series of diverse factors [7] which influenced the evolution of the disease as well as the treatment outcome. While these results led to new therapeutic approaches [8] which generated better treatment outcomes even in metastatic stages [9,10], cancer is still one of the leading causes of death in the EU and USA [11,12].

Several extensive investigations evaluated the existence of a relationship between the blood group and prognosis for patients diagnosed with malignancies, but even until now, this association remains unclear [13,14,15,16]. However, solid results linking the impact of the individual’s blood group on a certain malignancy risk came from recently published studies such as the one of Yang X. et al. [14] and Kakasava K. et al. [17]

Head and neck squamous cell carcinomas (HNSCC) are the sixth most common human malignancy worldwide, with a continuous rising in incidence up to an estimated 30% increased incidence in 2030 [18]. High mortality risk factors such as smoking, alcohol consumption and viral infections (e.g., EBV, HPV) are incriminated in the development of HNSCC [19].

Considering previously published data assessing the relative risk of head and neck cancers among different blood groups [20,21], we conducted an analysis to check whether ABO blood group impacts the susceptibility or protection against different types of head and neck cancers.

## 2. Materials and Methods

We conducted a retrospective analysis on cancer-diagnosed patients admitted at “Prof. Dr. Alexandru Trestioreanu” Institute of Oncology from Bucharest between January 2013 and February 2021. Patients’ medical charts were evaluated and besides demographic data (e.g., gender, age), the ABO blood group and primary site head and neck cancer diagnosis were extracted.

Electronic records were scraped using Python version 3 [22].

Patients were divided in seven head and neck primary malignancy subsites: hypopharynx, larynx, lip, nasopharynx, oral cavity, oropharynx, other, according to ICD-10 coding system [23]. For each group, the existence of a link between primary malignancy diagnosis and blood group was evaluated.

For statistical analysis, we used Chi-square test in R Studio [24] to check whether an association was found between cancer diagnosis and blood group. *p*-values lower than 0.05 were considered as significant. Exploratory analysis was conducted using ggplot2 and mosaic libraries from R Studio.

## 3. Results

We had an initial enrollment of 61,899 patients, and after excluding the patients for which the blood group was missing from their medical charts, 19,626 patients remained. Regarding demographic data, the mean patient age was 64 (±22) years, whilst the gender stratification resulted in 5773 males and 13,853 females. The most common blood group was A (44.39%) followed by 0 (33.64%), B (17.28%), and AB (4.69%). Selecting for primary head and neck cancer, 543 patients were eligible for analysis.

A total of 121 primary cancer sites were in the oropharynx (6.63%), 121 in the oral cavity (22.29%), and 86 in the larynx (15.84%). The most common blood group was A (245, 45.12%) followed by 0 (160, 24.66%), B (110, 20.26%), and AB (28, 5.16%) (Table 1).

**Figure 1:**
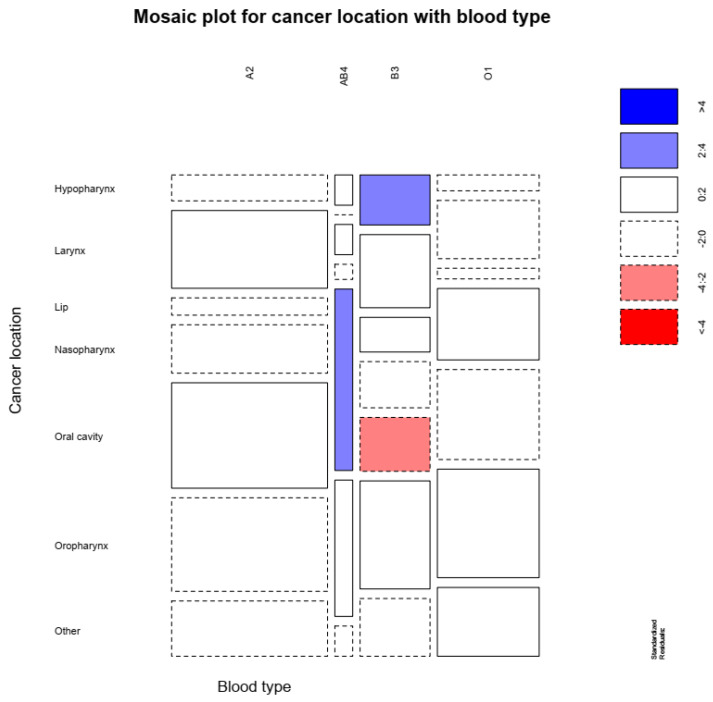
Higher hypopharynx cancer was associated with B group, oral cavity cancer was associated with a lower risk in patients with B group, while AB patients had a higher risk for oral cavity cancer.

Our study results, as presented in Figure 1, reveal that hypopharyngeal cancer was associated with the B group, oral cavity cancer was associated with a lower risk in patients with B group, while AB patients had a higher risk for oral cavity cancer (χ^2^ = 36.136, df = 18, *p* = 0.007).

## 4. Discussion

Although the first data regarding ABO changes in tumors were reported in 1930, only decades later were changes in the expression of blood group antigens reported in HNSCCs [25]. Regarding the blood group antigens, Hakomori et al. [26] reported that they are mostly expressed in endodermal epithelial cells, coincidence or not, the primary site of most human cancers. Research never ceased to continue in this field, and, more recently, in 2020, a prospective trial [27] on ABO(H) antigens conducted on 60 patients with oral cavity malignant and potentially malignant lesions, identified that the degree of loss of ABO(H) antigens in tissue specimens can be used as a marker of tumor stage of the patient. This conclusion resulted from the difference between poorly and moderately differentiated oral cavity carcinomas compared to well-differentiated carcinomas regarding the antigen reactivity.

The studies of Jaleel B. et al. [28] and Singh K. et al. [20] conducted on consistent populations of head and neck cancer patients assessed the relative risk of various HNSCCs amongst different blood groups. After data analysis, both studies were consistent in concluding that precursor H antigen, a protective factor against oral cancer, transformation to A and B antigens increases the risk of developing oral cancer and that O blood group have the highest H antigen amount and thus the lowest rates of oral cancer amongst the subjects with this blood group. Two years later, another study’s [17] results were published, reporting that on a population of 195 HNSCC patients group A showed the highest potential of developing this malignancy, compared to the other blood groups. Furthermore, in the Singh K. et al. [20] study, blood group A was found to be a potential risk factor for the development of salivary gland and esophageal cancer, whereas group B was found to be a potential risk factor for laryngeal cancer.

The association between nasopharyngeal carcinoma (NPC) and ABO blood groups has been previously studied, but the statistical data analysis resulted in conflicting findings. Initially, studies focused on the role of the ABO blood group as a risk factor for developing NPC. Therefore, while the study of Seow et al. [29] reported no association between ABO blood group and NPC, decades later, Turkoz et al. [30] found that the ABO blood group was related to NPC susceptibility. From the results reported in the latter study, blood type A was related to an increased risk of developing NPC, compared to blood group O, which showed a protective effect. More recent data regarding NPC came from China, where research focused more on the prognostic impact of ABO blood group on NPC. The authors of the initial studies linked the contradictory conclusions of their studies to the area-specific population and to geographic variations worldwide [31,32,33]. However, two recent studies [31,33] focusing on the population from Southeast China failed to reach a consensus regarding the prognostic value of ABO blood group on NPC. Moreover, genome-wide association studies [34,35,36] failed to link ABO blood groups to NPC risk. It is already widely accepted that lymphocyte counts are important prognostic factors for cancer patients [37,38,39,40]. The study of Zhang Y et al. [32] is the only one to evaluate the link between the ABO blood group, tumor stage and blood lymphocyte counts. The authors found that regarding the overall survival (OS) for locally advanced NPC patients with high lymphocyte levels, compared to non-A subgroups, the A blood group patients had better rates. A meta-analysis by Shao-wu Jing showed that, in Chinese patients, blood group O was associated with a lower incidence of NPC, while group A had no correlation with NPC [41].

The relationship between laryngeal cancer incidence and ABO blood group was previously studied. Three Polish studies [40,42,43,44] published between 1992 and 2000 failed to find a link between laryngeal cancer and ABO blood group. In the studies from Konieczna et al. [42] and Pyd et al. [43], blood group A2 was significantly more frequent in patients with glottic carcinoma, compared to the healthy population or to the other laryngeal subsites. Later on, in 2000, Nowinska et al. [44] failed to find a significant difference among ABO blood groups between laryngeal cancer patients and healthy individuals. More recently, published studies [45,46] also failed to reach a consensus on the relationship between ABO blood groups and laryngeal cancer. The study of Jin T. [46] et al. is the one with the largest cohort (1477 patients) and which showed that the ABO blood group was an independent prognostic factor for patients with laryngeal carcinoma. Therefore, patients with blood group O diagnosed with laryngeal carcinoma had almost 10% shorter 3-, 5-, and 10-year OS rates compared to the median OS of the patient population. The limited number of patients enrolled, the lack of strong statistical data, and being single-center studies are the main limitations that explain the current conflicting results for studies evaluating the prognostic value of the ABO blood group in laryngeal cancers.

The contradictory results regarding the relationship between ABO blood groups and HN cancers are reported for other sites also. In 2014, a study on 203 differentiated thyroid cancer patients, a lower risk was found for those with blood group B when compared to blood group O [47]. Five years later, another study [48] on thyroid cancer patients reported no significant association between this malignancy and ABO blood groups. However, in this later study, data analysis resulted in higher rates of extrathyroidal extension and advanced stage disease in B blood groups, compared to their non-B blood group counterparts.

## 5. Conclusions

Alongside the current literature data, our study’s results show that different blood groups are associated with different sites where head and neck cancer can arise. In our study, blood groups were found to be an important prognostic factor for tumor development. We identified that B blood antigen presence acts different according to the tumor site. For hypopharyngeal cancer, blood group B is associated with an increased incidence, whereas, for oral cavity, it acts as a protective factor, thus being linked to a lower incidence rate. Additionally, our study found that A blood antigen presence increases the risk of oral cavity cancer development, independent of B blood antigen presence. However, further investigations, including other factors such as environmental, demographic, and behavioral parameters, are still needed in order to corelate blood groups to the susceptibility or protection against different types of cancers.

## Figures and Tables

**Table 1:** Contingency table for H&N cancer localization with blood group.

Localization			Blood Type N (%)		
	O	A	AB	B	N (%)
Hypopharynx	6 (3.75)	15 (6.12)	2 (7.14)	13 (11.82)	36 (6.63)
Larynx	22 (13.75)	45 (18.37)	0 (0)	19 (17.27)	86 (15.84)
Lip	4 (2.5)	10 (4.08)	2 (7.14))	9 (8.18)	25 (4.6)
Nasopharynx	27 (16.88)	28 (11.43)	1 (3.57)	12 (10.91)	68 (12.52)
Oral cavity	34 (21.25)	61 (24.90)	12 (42.86)	14 (12.73)	121 (22.29)
Oropharynx	41 (25.63)	54 (22.04)	9 (32.14)	28 (25.26)	132 (24.31)
Other	26 (16.25)	32 (13.06)	2 (7.14)	15 (13.64)	75 (13.81)
Total	160 (29.47)	245 (45.12)	28 (5.16)	110 (20.26)	543 (100)

## Data Availability

Data is available upon request.
